# The use of intracardiac echocardiography catheters in endocardial ablation of cardiac arrhythmia: Meta‐analysis of efficiency, effectiveness, and safety outcomes

**DOI:** 10.1111/jce.14367

**Published:** 2020-01-30

**Authors:** Masahiko Goya, Diana Frame, Larry Gache, Yoko Ichishima, Daiane Oliveira Tayar, Laura Goldstein, Stephanie Hsiao Yu Lee

**Affiliations:** ^1^ Department of Cardiovascular Medicine Tokyo Medical and Dental University Tokyo Japan; ^2^ Real World Evidence CTI Clinical Trial & Consulting Covington Kentucky; ^3^ Medical Affairs Johnson & Johnson K.K. Tokyo Japan; ^4^ Health Economics & Market Access Johnson & Johnson Medical São Paulo Brazil; ^5^ Health Economics & Market Access Johnson & Johnson Medical Devices Irvine California; ^6^ Health Economics & Market Access Johnson & Johnson Medical Asia Pacific Singapore Singapore

**Keywords:** arrhythmia, catheter ablation, ICE, intracardiac echocardiography, intracardiac ultrasound

## Abstract

**Aims:**

The optimal use of intracardiac echocardiography (ICE) may reduce fluoroscopy time and procedural complications during endocardial ablation of cardiac arrhythmias. Due to limited evidence in this area, we conducted the first systematic literature review and meta‐analysis to evaluate outcomes associated with the use of ICE.

**Methods and Results:**

Studies reporting the use of ICE during ablation procedures vs without ICE were searched using PubMed/MEDLINE. A meta‐analysis was performed on the 19 studies (2186 patients) meeting inclusion criteria, collectively representing a broad range of arrhythmia mechanisms. Use of ICE was associated with significant reductions in fluoroscopy time (Hedges' *g* −1.06; 95% confidence interval [CI] −1.81 to −0.32; *P* < .01), fluoroscopy dose (Hedges' *g* −1.27; 95% CI −1.91 to −0.62; *P* < .01), and procedure time (Hedges' *g* −0.35; 95% CI −0.64 to −0.05; *P* = .02) vs ablation without ICE. A 6.95 minute reduction in fluoroscopy time and a 15.2 minute reduction in procedure time was observed between the ICE vs non‐ICE groups. These efficiency gains were not associated with any decreased effectiveness or safety. Sensitivity analyses limiting studies to an atrial fibrillation (AF) only population yielded similar results to the main analysis.

**Conclusion:**

The use of ICE in the ablation of cardiac arrhythmias is associated with significantly lower fluoroscopy time, fluoroscopy dose, and shorter procedure time vs ablation without ICE. These efficiency improvements did not compromise the clinical effectiveness or safety of the procedure.

## 1 INTRODUCTION

Atrial fibrillation (AF) and other cardiac arrhythmias affect more than 33 million individuals worldwide and are a major cause of stroke, heart failure, and death.^1^ Although established as an effective and safe treatment, ablation carries a small risk of complications such as thromboembolism, phrenic nerve injury, pulmonary vein stenosis, cardiac tamponade, and esophageal fistula.^2^ Catheter ablation procedures may also require prolonged fluoroscopic guidance, which exposes the patient, operator, and laboratory staff to significant levels of radiation.^3^ Estimates suggest that an average fluoroscopy time of 1 hour during ablation increases a patient's lifetime risk of fatal cancer by up to 0.1%.^4^ It has been reported that cardiac electrophysiologists have an annual ionizing radiation exposure two to three times higher than that of diagnostic radiologists, which translates to a cumulative risk of one additional cancer diagnosis for every 100 exposed individuals following a full professional career.^5^, ^6^

Preprocedural computed tomography (CT) or magnetic resonance imaging (MRI) can be used to visualize the location of the left atrium, pulmonary veins, and surrounding structures before ablation. Preprocedural images can also be merged into three‐dimensional electroanatomical mapping (EAM) systems to visualize the esophagus and its location along the posterior wall of the left atrium, and reduce radiation exposure during the ablation procedure.^7^ However, because of changes in patient position, esophagus location, and/or cardiac rhythm, use of CT or MRI images obtained pre‐procedurally to guide catheter manipulation may not accurately reflect the actual cardiac anatomy.^8^, ^9^, ^10^ Despite advances in EAM and catheter technology, most complex procedures still utilize prolonged fluoroscopic guidance which exposes the patient, operator, and laboratory staff to radiation.^3^

Intracardiac echocardiography (ICE) has multiple real‐time applications during catheter ablation, including the ability to guide transseptal puncture, visualize the location of the esophagus, provide guidance of cardiac anatomy, and detect microbubbles as a result of overheating, thereby preventing complications.^11^, ^12^ Optimal use of ICE may reduce fluoroscopy time and procedural complications; however, there is limited evidence comparing the use of ICE with procedures dependent on fluoroscopy or other imaging modalities to procedures that do not use ICE. Published evidence comparing ablation with ICE vs ablation without ICE have also not been analyzed in a meta‐analysis comparing clinical endpoints to date. Therefore, a systematic literature review and meta‐analysis were conducted to evaluate the use of ICE for real‐time imaging during endocardial ablation of various cardiac arrhythmias.

## 2 METHODS

We conducted a systematic literature review following typical best practices, including the use of a prospective protocol specifying search terms and study eligibility.^13^ A statistical analysis plan was also prepared *a priori*.

### 2.1 Search strategy

We searched PubMed/MEDLINE to identify studies published in English between 1 January 1996 and 31 October 2018, that assessed the use of ICE during the ablation of cardiac arrhythmias vs ablation without ICE. The electronic search was supplemented by manually searching reference lists of recent review articles. Search terms used in the literature search are detailed in Table S1.

### 2.2 Study eligibility

Studies were screened for eligibility using a two‐step process and prespecified criteria. During “level I” review, studies were screened for eligibility by a single reviewer, based on their title and abstract. Potentially eligible studies with an eligible patient population and some indication that ICE was evaluated were reviewed in full text. During “level II” screening, full‐text articles were reviewed for use of the technology of interest and for eligible comparative data. At level II screening, two reviewers independently assessed each study for fit with the selection criteria.

Included studies were required to be in English‐language, comparative (randomized or nonrandomized, including retrospective comparisons), and with at least 10 patients undergoing endocardial ablation with either sensor‐based (SOUNDSTAR® Catheter, Biosense Webster, Inc) or non‐sensor‐based (eg, AcuNav^TM^ Catheter, Biosense Webster, Inc; Ultra ICE^TM^ Catheter, Boston Scientific; or ViewFlex^TM^ Catheter, St. Jude Medical) catheters compared to each other, or to ablation procedures without the use of ultrasound. We deliberately included reports of ablation for any form of cardiac arrhythmia to broadly assess the use of ICE according to real‐world clinical practice. Studies were excluded if they did not include a procedure of interest, did not use real‐time guidance during ablation (eg, preprocedural evaluation of left atrial appendage thrombus only), or did not report any outcomes of interest (ie, extractable data for ICE and comparator for at least one efficiency, effectiveness, or safety outcome).

### 2.3 Data extraction and quality assessment

For the preparation of the meta‐analysis data set, all data elements were extracted by one reviewer and confirmed by a second reviewer. Key study, patient, and treatment characteristics were captured from each study using a standard template, and studies were assessed for quality using the Oxford Level of Evidence Centre for Evidence‐Based Medicine (CEBM) level of evidence.^14^ Randomized clinical trials (RCTs) were considered level 2, except where the ICE comparison was confounded by inclusion of some use of ICE in the control arm or by other imaging techniques which varied between groups. These “downgraded” RCTs were considered level 3 evidence, along with prospective non‐randomized comparative studies. Retrospective non‐randomized comparative studies were considered level 4 evidence. Efficiency, effectiveness, and safety outcomes were extracted from all eligible studies, as available. Safety events were divided into venous access vs all other peri‐procedural complications, to isolate the setup‐related outcomes from those associated with the ablation itself.

### 2.4 Data synthesis and analysis

The primary endpoint for the meta‐analysis was fluoroscopy time. Secondary outcomes were fluoroscopy dose, procedure time, acute procedure success, peri‐procedural complications, and freedom from arrhythmia at 6 months follow‐up or longer.

#### 2.4.1 Outcome study measures

Intention‐to‐treat results were extracted for all binary outcomes, with between‐group effect sizes compared using a risk ratio (RR). Continuous and time outcomes were compared using standardized mean difference (with Hedges' *g* adjustment). The standardized mean difference allows the analysis of studies assessing the same outcome, but with different outcome definitions, data reporting formats, or measurement scales. Absolute Hedges' *g* values <0.2 indicate a small effect, 0.5 a medium effect, and >0.8 a large effect.^15^ For this study, negative Hedges' *g* values are desirable. Mean difference (MD; in minutes) analysis was also performed on fluoroscopy and procedure time outcomes. In studies with three arms, we chose the two arms with the cleanest ICE vs non‐ICE comparison (eg, a group including ICE for imaging during the ablation procedure vs a group without ICE, but with otherwise similar mapping and ablation techniques). Since the choice was not always clear‐cut, we also performed a sensitivity analysis using the alternate comparator arm.

#### 2.4.2 Analyses

Because heterogeneity was expected among studies, the main analyses were performed with random‐effects inverse variance weighting models, as recommended by Fleiss et al^16^ using both the DerSimonian‐Laird (DL) method^17^ and Hartung‐Knapp‐Sidik‐Jonkman (HKSJ) method.^18^, ^19^, ^20^ The DL method is currently considered the standard for random‐effects models. However, the HKSJ method has been found to provide more consistently adequate error rates, especially when the number of studies is small and there is moderate or substantial heterogeneity, and thus is increasingly accepted as a more appropriate method.^21^ Since the acute procedure success and peri‐procedural complications outcomes showed sparse data for events or non‐events, they were estimated using a random‐effects Mantel‐Haenszel (MH) risk ratio.

Sensitivity analyses were performed to understand the robustness of the initial effect size estimates and to assess potential sources of heterogeneity among studies. When data permitted, the following sensitivity analyses were conducted: effect size calculation using the HKSJ method, removal of outlying and statistically influential studies, imputation of non‐reported study means and/or standard deviations (SDs), and use of an alternate comparator arm. For fluoroscopy time, sensitivity associated with the use of a sensor‐based ICE catheter (SOUNDSTAR® Catheter) was also performed. In particular, extensive post‐hoc sensitivity analyses were conducted for studies restricted to an AF population across all endpoints of interest, after our systematic review revealed that a majority of reports focused on this arrhythmia type. Doing so eliminated all studies with a pediatric cohort, as well as those with substrates distinctive from AF.

Data manipulation and statistical analyses were performed using SAS Software, Version 9.4 and the R meta‐package, Version 4.9‐4. All *P*‐values were two‐sided, and values ≤.05 were considered statistically significant.

## 3 RESULTS

**Figure 1:**
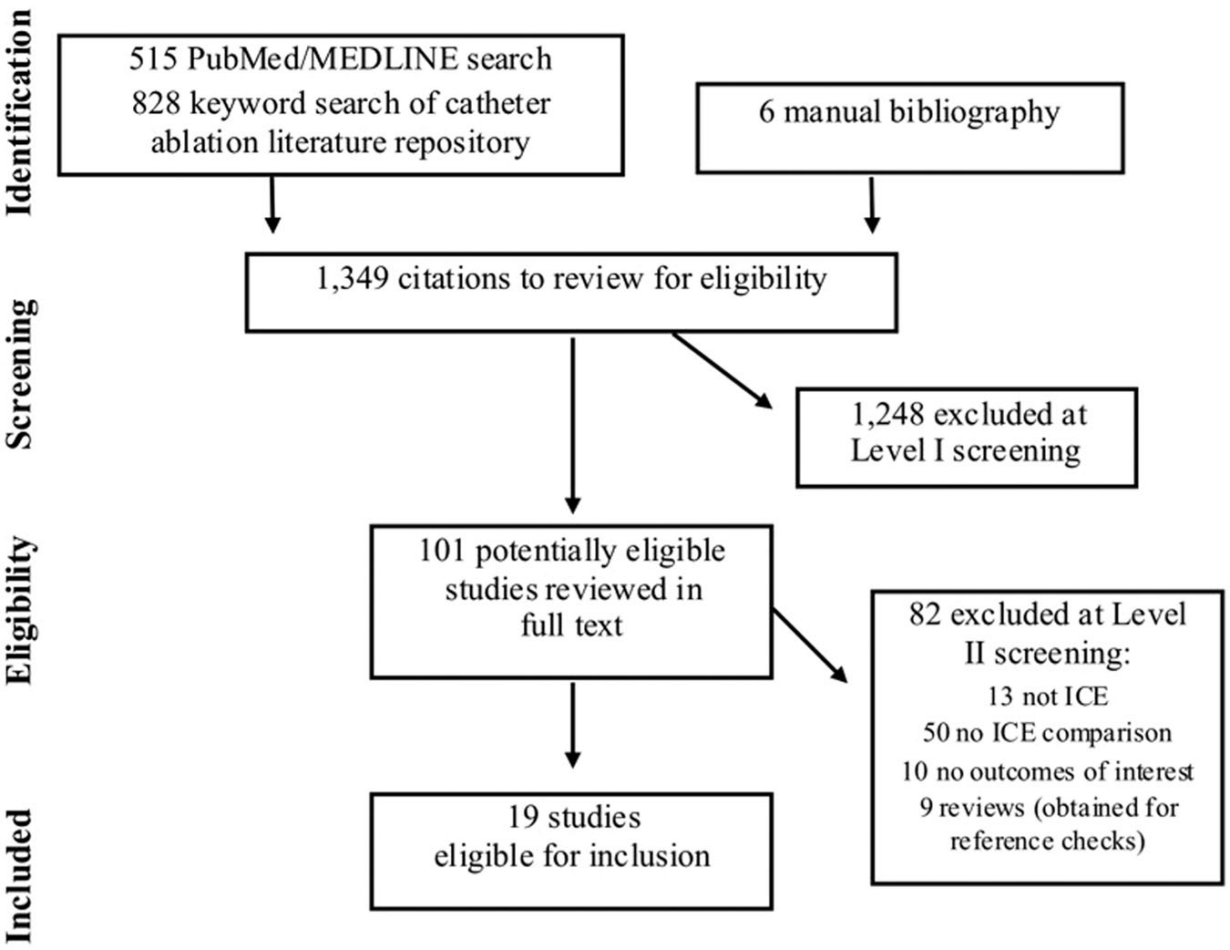
Flow diagram of the stages of the systematic literature search. ICE, intracardiac echocardiography

Our initial search retrieved 1349 articles (Figure 1). After exclusion by title and abstract (“Level I” screening), 101 potentially eligible studies were reviewed in full text. Eighty‐two articles were excluded during the “Level II” screening. The most common reasons for exclusion were studies that did not use ICE during the procedure or did not include an ICE vs no ICE comparison.

### 3.1 Study characteristics

Nineteen studies with a total sample size of 2186 patients were included in the main analysis, encompassing patients with any form of atrial or ventricular arrhythmia undergoing catheter ablation (see Table S2 for individual study details).^10^, ^22^, ^23^, ^24^, ^25^, ^26^, ^27^, ^28^, ^29^, ^30^, ^31^, ^32^, ^33^, ^34^, ^35^, ^36^, ^37^, ^38^ The characteristics of the included studies are summarized in Table 1. The overall mean age of patients in the included studies was 54.8 years, and 65.3% of patients were male. The majority of the studies were RCTs (11 studies) and included a strictly AF population (13 studies).

**Table 1:** Characteristics of studies included in the meta‐analysis

Study characteristic	Number of studies (N = 19)	Number of patients (N = 2186)
Year of Publication		
2009 and earlier	4	468
2010–2018	15	1718
Study design		
RCT	11	664
Prospective comparative, non‐RCT	1	37
Retrospective comparative, non‐RCT	7	1485
Oxford Level^a^		
Level 2 (RCT)	8	471
Level 3 (Prospective non‐RCT or downgraded RCT)	4	230
Level 4 (Retrospective non‐RCT)	7	1485
Arrhythmia type		
AF	13	1772
AFL	1	80
AVNRT	1	40
VT, PVC	1	16
Mixed	3	278
ICE technology		
Non‐sensor based ICE catheter (ie, AcuNav^TM^ Catheter)	12	1869
Sensor‐based ICE catheter (SOUNDSTAR® Catheter)	5	240
Other/mixed ICE	2	77
Comparator technology		
Electroanatomic mapping	8	786
Fluoroscopy	8	762
Other/mixed no ICE	3	638
Population age type		
Pediatric (average age <18)	2	158
Adult (average age ≥18)	17	2028

Abbreviations: AF, atrial fibrillation; AFL, atrial flutter; ICE, intracardiac echocardiography; AVNRT, atrioventricular nodal re‐entrant tachycardia; PVT, premature ventricular contractions; RCT, randomized controlled trial; VT, ventricular tachycardia.

^a^ Three RCTs were downgraded from level 2 to level 3 due to confounding/quality issues.^25^, ^30^, ^33^

### 3.2 Procedural efficiency outcomes

#### 3.2.1 Fluoroscopy time

**Figure 2:**
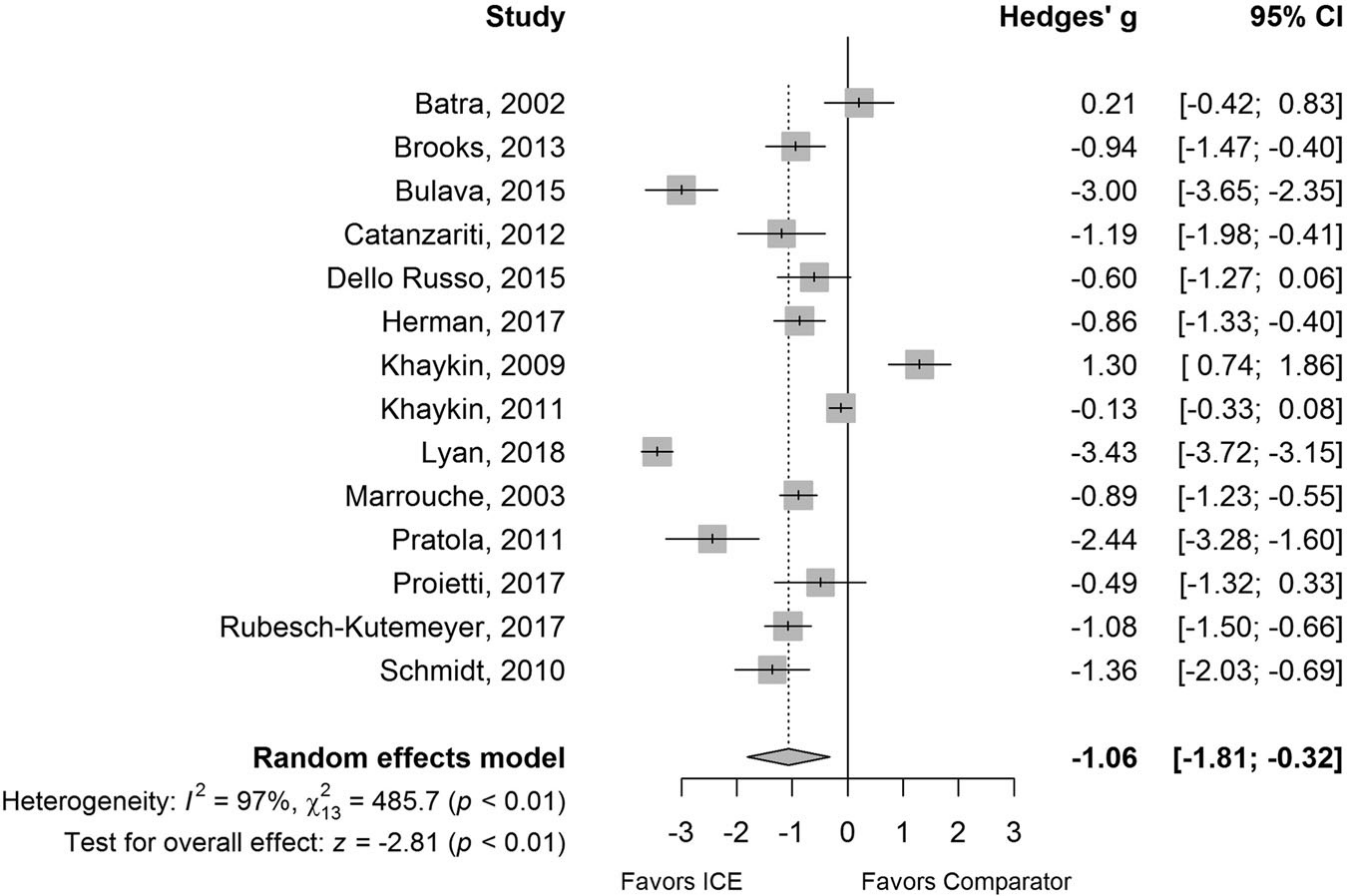
Forest plot of Hedges’ *g* analysis of fluoroscopy time (primary outcome) in meta‐analysis of the use of ICE vs comparator (no ICE) in catheter ablation of cardiac arrhythmias. CI, confidence interval; ICE, intracardiac echocardiography

Data for fluoroscopy time were available from 14 studies, with significant heterogeneity among studies (I^2^ = 97%).^10^, ^11^, ^22^, ^24^, ^25^, ^26^, ^27^, ^28^, ^29^, ^30^, ^31^, ^35^, ^37^, ^38^ Compared to ablation without ICE guidance, the use of ICE was associated with a significant decrease in fluoroscopy time (primary outcome) (Hedges' *g* −1.06; 95% CI −1.81 to −0.32; *P* < .01) (Figure 2). This amounted to an average reduction of 6.95 minutes (MD −6.95; 95% CI −11.25 to −2.66; *P* < .01) (Figure S1A). The funnel plot and Egger's test of asymmetry (*P* = .9670) suggested no publication bias or small‐study effects (Figure S1B).

The significant reduction in fluoroscopy time demonstrated in the main analysis was robust in all sensitivity testing, including studies with only AF patients (Hedges' *g* −1.25; 95% CI −2.14 to −0.36; *P* < .01).^10^, ^11^, ^24^, ^26^, ^27^, ^28^, ^29^, ^30^, ^31^, ^37^, ^38^ (Table S3). In addition, analyses that removed an outlying and influential study (*P* < .01),^31^ included studies with imputed means and/or SD values (*P* < .01),^32^, ^34^, ^36^ used an alternate group for 3‐arm studies (*P* = .04),^10^, ^11^, ^29^ used the HKSJ method (*P* < .01), or used only sensor‐based ICE catheters (*P* = .02),^10^, ^24^, ^35^ all remained statistically significant. For sensor‐based ICE catheters, mean difference analysis demonstrated a 12.74 minute reduction in fluoroscopy time.

#### 3.2.2 Fluoroscopy dose

**Figure 3:**
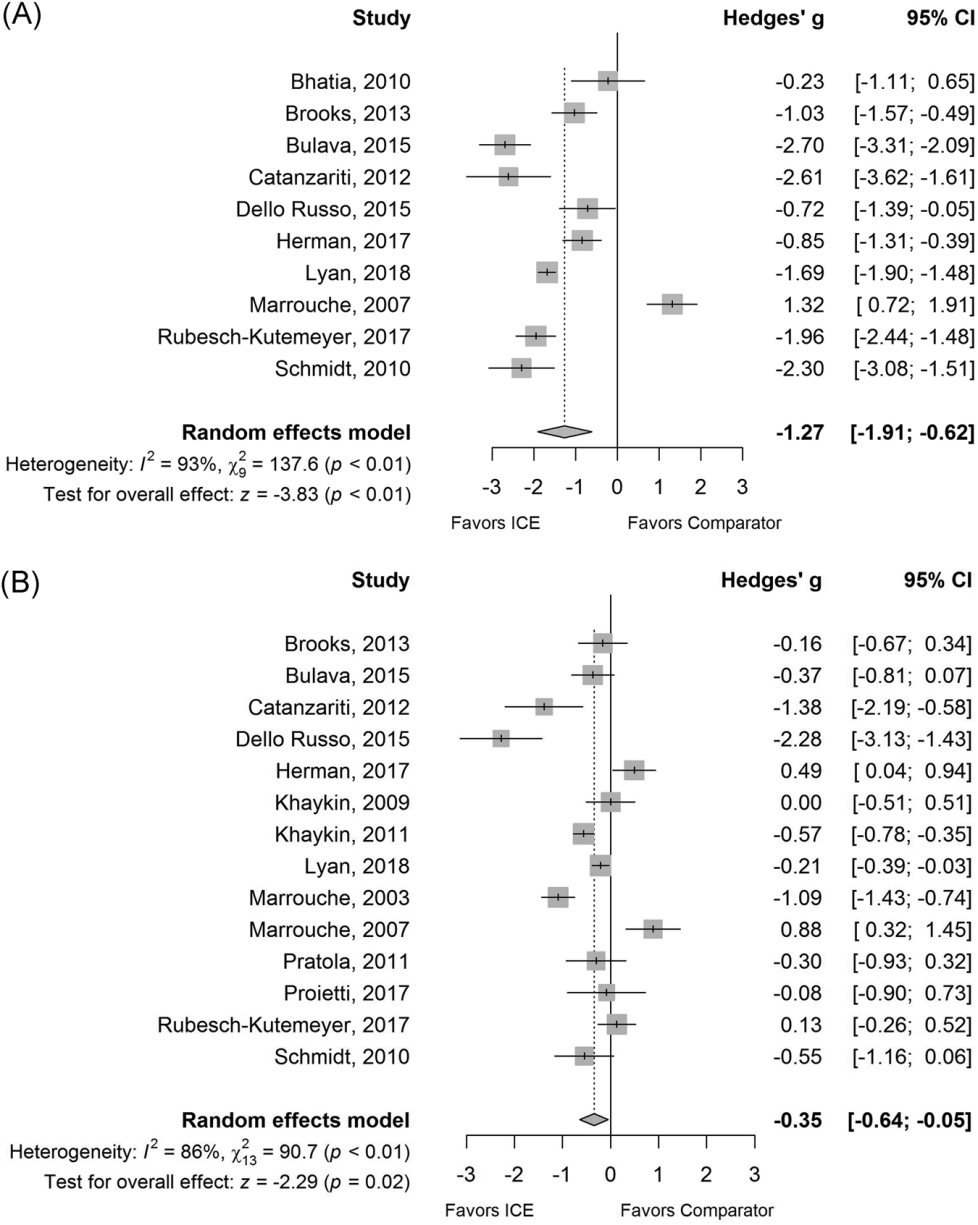
Forest plot of Hedges’ *g* analysis of A, fluoroscopy dose and B, procedure time in the meta‐analysis of the use of ICE vs comparator (no ICE) in catheter ablation of cardiac arrhythmias. CI, confidence interval; ICE, intracardiac echocardiography

Ten studies reported fluoroscopy dose data, with significant heterogeneity among studies (I^2^ = 93%) and no evidence of publication bias or small study effects.^23^, ^24^, ^25^, ^26^, ^27^, ^28^, ^31^, ^33^, ^37^, ^38^ Use of ICE in ablation procedures showed significant reductions in fluoroscopy dose vs ablation without the use of ICE (Hedges' *g* −1.27; 95% CI −1.91 to −0.62; *P* < .01) (Figure 3A). This result was robust, with similar effect sizes reported across various sensitivity analyses, including the removal of one outlying and influential study *(P* < .01),^33^ inclusion of two studies with imputed means and/or SD values (*P* < .01),^32^, ^34^ and use of the HKSJ method on the full data set (*P* = .01). Similar results were found when limiting studies to an AF population (Hedges' *g* −1.32; 95% CI −2.04 to −0.59; *P* < .01) (Table S4).

#### 3.2.3 Procedure time

Data for procedure time were available from 14 studies, with significant heterogeneity among studies (I^2^ = 86%) and no evidence of publication bias or small study effects.^10^, ^11^, ^24^, ^25^, ^26^, ^27^, ^28^, ^29^, ^30^, ^31^, ^33^, ^35^, ^37^, ^38^ A significant decrease in procedure time was associated with ablation procedures using ICE vs those without (Hedges' *g* −0.35; 95% CI −0.64 to −0.05; *P* = .02) (Figure 3B). This corresponded to an average reduction of 15.2 minutes (MD −15.2; 95% CI −26.40 to −4.0; *P* < .01) (Figure S2). These results were consistent in a sensitivity analysis restricting studies to an AF population (Hedges' *g* −0.43; 95% CI −0.74 to −0.13; *P* < .01). Other sensitivity analyses including use of the HKSJ method (*P* = .10), removal of one outlying and influential study (*P* = .09),^27^ inclusion of two studies with imputed means and/or SD values (*P* = .09),^23^, ^34^ and use of an alternate group for 3‐arm studies (*P* = .07),^10^, ^11^, ^29^ were directionally similar but failed to reach statistical significance (Table S4).

### 3.3 Effectiveness outcomes: acute success and freedom from arrhythmia

#### 3.3.1 Acute success

Thirteen studies were included in the analysis,^10^, ^22^, ^25^, ^26^, ^27^, ^28^, ^31^, ^32^, ^33^, ^34^, ^35^, ^36^, ^38^ 11 of which reported 100% acute success in one or both arms.^10^, ^22^, ^25^, ^26^, ^27^, ^28^, ^31^, ^33^, ^34^, ^35^, ^36^ Therefore, there was little heterogeneity among studies (I^2^ = 30%). Acute success was high in all studies and not significantly different in ablation procedures using ICE vs those without (RR 1.01; 95% CI, 0.99‐1.02; *P* = .43) (Figure S3A). Sensitivity performed on AF only patients yielded consistent results (RR 1.00; 95% CI, 0.99‐1.01; *P* = .86).

#### 3.3.2 Freedom from arrhythmia

Eleven studies reported freedom from arrhythmia outcomes, with no observable heterogeneity among studies (I^2^ = 0%).^10^, ^11^, ^24^, ^25^, ^26^, ^27^, ^30^, ^31^, ^33^, ^37^, ^38^ All studies for this outcome were in the setting of AF. The use of ICE was not associated with a change in freedom from arrhythmia compared to ablation without the use of ICE (RR 1.04; 95% CI, 0.97‐1.11, *P* = .24) (Figure S3B). No outlying or influential studies were identified to include in a sensitivity analysis. Sensitivity analyses using an alternate group for the 3‐arm study^10^ (*P* = .33) and using the HKSJ difference method (*P* = .23) did not demonstrate any significant differences in freedom from arrhythmia between groups (Table S5).

### 3.4 Safety outcome: peri‐procedural complications (excluding venous access)

Six studies reported zero (0) peri‐procedural complications in one or both arms^10^, ^26^, ^27^, ^28^, ^30^, ^36^ and there was no observable heterogeneity among the 13 studies included in the analysis (I^2^ = 0%).^11^, ^22^, ^24^, ^26^, ^27^, ^28^, ^30^, ^31^, ^32^, ^34^, ^36^, ^37^, ^38^ A nonsignificant decrease in complications was demonstrated in ablations with the use of ICE vs no ICE (RR 0.66; 95% CI, 0.42‐1.05, *P* = .08) (Figure S3C). In the sensitivity analysis that included an alternate group for 3‐arm studies,^10^, ^11^ a significantly lower RR for peri‐procedural complications was demonstrated between the ICE vs no ICE groups (RR 0.50; 95% CI, 0.30‐0.83, *P* < .01). There was no significant difference among the groups when restricting studies to only AF ablations (*P* = .24) (Table S5).^10^, ^11^, ^22^, ^24^, ^26^, ^27^, ^28^, ^30^, ^31^, ^32^, ^34^, ^36^, ^37^, ^38^

### 3.5 Safety outcome: venous access complications

Five studies reported zero (0) venous access complications in one or both arms^10^, ^27^, ^31^, ^37^, ^38^ and there was no observable heterogeneity among the 10 studies included in the analysis (I^2^ = 0%).^10^, ^24^, ^27^, ^28^, ^31^, ^32^, ^34^, ^36^, ^37^, ^38^ Event rates were not statistically different between groups but trended in favor of non‐ICE (RR 1.93; 95% CI, 0.81‐4.60; *P* = .14) (Figure S3D). No studies with an alternate group for 3‐arm studies were available for sensitivity analysis, and limiting reports to an AF population yielded nonsignificant results consistent with the main analysis (*P* = .21) (Table S6).^10^, ^24^, ^27^, ^31^, ^37^, ^38^

## 4 DISCUSSION

To the best of our knowledge, this is the first meta‐analysis comparing ICE‐guided ablation with procedures using fluoroscopy or other mapping/navigation systems that do not use ICE. Our study highlights that catheter ablation of a range of cardiac arrhythmias using real‐time imaging with ICE is associated with significant reductions in fluoroscopy time (primary outcome), fluoroscopy dose, and procedure time compared with ablation without the use of ICE. These reductions were not accompanied by an evident decrease in effectiveness or safety.

Prolonged fluoroscopic guidance during ablation exposes the patient to significant levels of radiation.^3^ Radiation exposure is associated with acute and subacute skin injury, malignancies, cataracts, thyroid dysfunction, and other diseases.^4^, ^39^, ^40^, ^41^, ^42^, ^43^ Because of these potential risks and the linear relationship between radiation dose and increased risk of future malignancy (ie, no dose of radiation is considered safe), the underlying principle of radiation exposure states that radiation dose must be as low as reasonably achievable (ALARA).^44^ Certain patient groups have been identified that are more vulnerable to radiation risks. For example, obese patients were shown to require nearly three times the amount of radiation exposure than what is required for nonobese patients.^45^ Radiation risks are also higher for children and pregnant women.^46^, ^47^

Operators and staff who perform many ablation procedures over time accumulate significant exposure to radiation; therefore, these individuals are also highly susceptible to the risks associated with heavy fluoroscopy use, which may be even greater than that experienced by patients.^48^ Brain tumors, breast cancer in female cardiologists, and cataracts have been reported in interventional cardiologists and staff potentially due to their increased radiation exposure.^49^, ^50^, ^51^ In addition, a higher prevalence of orthopedic injuries have been reported in interventional cardiologists, who wear lead apparel to protect themselves from radiation, compared to noninterventional cardiologists.^52^ Studies indicate that over one‐third of interventional cardiologists with spinal complaints miss work due to spine problems.^53^, ^54^

Because of the risks of radiation, technological strategies including the use of ICE have been developed to reduce hazardous exposure to patients and operators. This meta‐analysis demonstrates that the use of ICE during ablation results in significantly lower duration and a dose of fluoroscopy compared to ablation without ICE. The planned sensitivity analyses for these endpoints found the results to be robust. Ablation procedures using ICE were associated with a 6.95 minute shorter fluoroscopy time compared to ablation without the use of ICE. Significant reductions in fluoroscopy time with a sensor‐based ICE catheter were also observed in a mean difference analysis demonstrating a 12.74 minute reduction.^10^, ^22^, ^35^ Increased safety through fluoroscopy time and dosage reduction with ICE may minimize radiation risks for patients, operators, and staff. Correspondingly this may also diminish the need for prolonged lead protection for interventional cardiologists and staff, helping to reduce occupational orthopedic injuries.

Real‐time guidance and visualization of cardiac anatomy is critical for procedural efficiency in catheter ablation procedures. ICE provides additional information to the operator beyond angiography or other forms of imaging, by not only producing accurate procedural imaging of the cardiac anatomy but also providing guidance for the trans‐septal puncture to ensure safe and reliable access to the left atrium.^12^ This review demonstrates that the use of ICE during endocardial ablation resulted in significant reductions in procedure time (15.2 minutes) vs ablation without the use of ICE. Similar effect sizes were found across various sensitivity analyses, though not all reached statistical significance. Variations in reported procedure times across institutions/operators, and the potential learning curve associated with the use of ICE, complicate the assessment of procedure time. Further investigation is warranted to determine the precise factors that influence procedural efficiency with ICE and to shed light on any factors and mechanisms contributing to shorter procedure time.

In this study improved efficiency (ie, reductions in fluoroscopy and procedure times) using ICE did not compromise effectiveness. Based on the main analyses, freedom from arrhythmia was not significantly different for ICE vs comparators (*P* = .24). In clinical practice, the use of ICE throughout the ablation procedure varies from physician to physician. Studies meeting the selection criteria for our meta‐analysis provided limited methodological descriptions on their use of ICE; therefore, it is not clear how precisely ICE was used during ablation, and whether the full extent of potential benefits was realized. Regardless, hospitals or operators that do not routinely integrate CT/MRI into their ablation workflow should see a compelling value for the use of ICE.

Reductions in fluoroscopy and procedure times with ICE were also not accompanied by any evident increase in risk. In fact, non‐venous access complications trended lower for ICE vs comparators (RR 0.66, *P* = .08). A significantly lower RR was also found in the alternate‐arm sensitivity analysis (RR 0.50, *P* < .01). Beyond the transseptal puncture, ICE can provide benefits through different stages of catheter ablation, and may reduce the incidence of complications such as pericardial effusion, tamponade, thrombus formation, or pulmonary veins stenosis.^12^ Integration of ICE with EAM also allows for the direct visualization and improved guidance of anatomical structures, including esophageal imaging to prevent atrioesophageal fistula, and the detection of microbubbles that may indicate excessive catheter heating during radiofrequency ablation.^11^, ^12^ Venous access complications appeared numerically higher for ICE over the comparator. However, the difference between groups was not statistically significant (RR 1.93, *P* = 0.14). In our meta‐analysis, peri‐procedural complications were rare, with many studies reporting no events in one or both comparator arms. Due to such rarity, it is difficult to definitively assess a statistical difference in the rate of complications among procedures that use ICE vs those that do not. In a 2013 systematic review examining complications of catheter ablation in AF, the incidence of peri‐procedural complications was reported at 2.9%.^55^

Overall, sensitivity analyses of outcomes limited to AF ablation were consistent with findings from the main analyses, which broadly included other arrhythmia types (Table 2). In fact, most effect sizes were more pronounced when constrained to strictly AF studies. Atrial fibrillation is by far the most common sustained arrhythmia, and its various forms (ie, paroxysmal, persistent, permanent) require individualized ablation strategies. Despite these differences, ICE guidance should, in theory, enhance catheter ablations regardless of arrhythmia type. Indeed, ICE is currently utilized in clinical practice across a range of cardiac procedures, including atrial septal defect repair, left atrial appendage closure, and transcatheter aortic valve replacement. Although these interventions were outside the scope of our study, it would be of interest to understand how ICE inclusion impacts these procedures as part of future research.

**Table 2:** Summary of results for the main analysis and AF‐only sensitivity analyses

Outcome of interest	Main analysis (all arrhythmia types)		Sensitivity analysis (AF only)	
	Estimate^a^	*P*‐value	Estimate^a^	*P*‐value
Fluoroscopy time (Hedges’ *g*)	−1.06	<.01	−1.25	<.01
Fluoroscopy time (MD, min)	−6.95	<.01	−8.12	<.01
Fluoroscopy dose (Hedges’ *g*)	−1.27	<.01	−1.32	<.01
Procedure time (Hedges’ *g*)	−0.35	.02	−0.43	<.01
Procedure time (MD, min)	−15.2	<.01	−17.96	<.01
Acute success (RR)^b^	1.01	.43	1.00	.86
Peri‐procedural complications, excluding venous access (RR)^b^	0.66	.08	0.71	.24
Venous access complications (RR)^b^	1.93	.14	3.26	.21
Freedom from arrhythmia (RR)	1.04	.24	*Same – all studies in AF*	

Abbreviations: AF, atrial fibrillation; MD, mean difference; RR, risk ratio.

^a^ Unless otherwise stated, difference estimates are based on the DerSimonian‐Laird (DL) method.

^b^ Difference estimates were calculated using the Mantel‐Haenszel (MH) method.

An evaluation of the economic impact of adopting ICE in routine ablation procedures is another area of potential scientific interest. From a hospital perspective, efficiency gains resulting from a shorter procedure time may partially offset costs associated with the ICE catheter, especially given the premium placed on operating expensive electrophysiology labs and deploying teams of highly skilled healthcare professionals. From a payer perspective, the potential to reduce complication rates (for patients) and occupational hazards (for clinicians) may contribute to lower downstream treatment costs. However, it is difficult to generalize the determination of “cost‐effectiveness” from country to country. Each healthcare system is subject to diverse perspectives of value, influenced by current cultural and political factors, existing healthcare financing models, level of economic development, and local unmet medical needs. Robust and nuanced analysis is needed to further explore this topic.

## 5 LIMITATIONS

Our study included 19 publications in the first meta‐analysis comparing the endocardial ablation of any cardiac arrhythmia with ICE vs procedures using fluoroscopy or other mapping/navigation systems. However, there were several limitations to our work, many of which are inherent to any meta‐analysis. First, our systematic literature search only included English‐language published evidence indexed within PubMed/MEDLINE or discoverable by manual reference checks. Most of the included studies were conducted in North America and Europe. Second, we included comparative data from both randomized and non‐randomized studies due to the limited number of RCTs available, and among the included RCTs, three studies were downgraded from level 2 to level 3 due to confounding/quality issues. Third, the comparator group was broad and included EAM, fluoroscopy, or other/mixed imaging modalities without the use of ICE. Fourth, the time period of the included studies (>20 years) was also broad. Technological advances in ablation of arrhythmias have dramatically improved over the past 5 to 10 years. Procedures and techniques, as well as diagnostic and ablation catheters have become more sophisticated, which has led to improved safety, efficacy, and efficiency. Lastly, there was significant heterogeneity among studies in analyses examining efficiency outcomes (fluoroscopy time, fluoroscopy dose, and procedure time), which might reflect different study periods, hospital workflows, or individual operator skills. Nevertheless, there was no evidence of publication bias or small‐study effects, and the treatment effects were robust in a sensitivity analysis.

## 6 CONCLUSIONS

Ablation of various cardiac arrhythmias using ICE is associated with significant reductions in fluoroscopy time, fluoroscopy dose, and procedure time when compared to ablation without the use of ICE. These efficiency improvements did not appear to negatively impact clinical effectiveness or safety of the ablation procedure.

## AUTHOR CONTRIBUTIONS

Conception and design: DF, LG, YI, LG, and SL. Collection and assembly of data: DF and LG. Data analysis and interpretation, manuscript writing and final approval of manuscript: All authors.

## Supporting information

Supporting information
