# Supplementary material for: The use of intracardiac echocardiography catheters in endocardial ablation of cardiac arrhythmia: Meta‐analysis of efficiency, effectiveness, and safety outcomes
Source: J Cardiovasc Electrophysiol. 2020 Jan 30;31(3):664–73. doi: 10.1111/jce.14367 (PMC7078927; doi:10.1111/jce.14367)
Supplement: Supplementary file 1 — Supporting information [file JCE-31-664-s001.docx]

**Supplementary Material**

**Supplemental Table 1: Search strategy.**

| The initial literature search was conducted in the National Library of Medicine's PubMed database using the following terms, where "MeSH" indicates a MEDLINE Medical Subject Heading and "tiab" denotes a keyword to be sought in the title and/or abstract: |
| --- |
| 1. Catheter ablation[MeSH] OR "ablation" OR "cryo-ablation" OR cryoablation OR cryo-balloon OR cryoballoon OR "laser balloon" |
| 1. Arrhythmias, Cardiac[MeSH] OR "atrial fibrillation" OR "atrial flutter" OR tachycardia OR arrhythmia OR arrhythmias OR atrium OR atrial OR AFib[tiab] OR "pulmonary vein isolation" OR ventricular |
| 1. AcuNav OR CartoSound OR Soundstar OR UltraICE OR "Ultra ICE" OR "intracardiac ultrasound" OR "intracardiac echocardiography" OR "ICE-guided" OR "ICE"[tiab] |
| 1. #1 AND #2 AND #3, Limits: English, 1/11996-10/31/2018; NOT comment, editorial, news item, or narrative review |

Supplemental Table 2: Characteristics of Studies Included in the meta-analysis.

| **Study** | **Study Design** | **Total Patients**  **(N)** | **Cohorts Studied** | | **Arrhythmia Type** | **Ablation Energy Source** | **Mean Follow-Up*** | **Oxford**  **Level of Evidence** |
| --- | --- | --- | --- | --- | --- | --- | --- | --- |
|  |  |  | **ICE** | **No ICE** |  |  |  |  |
| Batra, 2002 | RCT | 40 | non-sensor-based ICE catheter (9F, 9MHz Boston Scientiﬁc Corp, with Panasonic Clear View ultrasound system)  n = 20 | fluoroscopy and intracardiac electrograms  n = 20 | AVNRT | RF | ICE:  30 ± 12 weeks  No ICE  48 ± 2 weeks | 2 |
| Bhatia, 2010 | Retrospective comparative non-RCT | 20 | CARTO-integrated, sensor based ICE catheter  (SoundStar) n = 20 | EAM (CARTO, CARTOMerge)  n = 10 | AF | RF | ICE 110 + 20 days  No ICE:  219 + 10 days | 4 |
| Brooks, 2013 | RCT | 60 | CARTO-integrated, sensor based ICE catheter  (SoundStar)  n = 30 | EAM (CARTO XP, Lasso)  n = 30 | AF  paroxysmal: n = 29  persistent: n = 23  long-standing persistent: n = 8 | RF | 13 ± 5 months | 2 |
| Bulava, 2015 | RCT | 80 | non-sensor-based ICE catheter (AcuNav) | Fluoroscopy, CARTO-3 | paroxysmal AF | RF | 12 months | 3^ |
| Catanzariti, 2012 | RCT | 30 | non-sensor-based ICE catheter (AcuNav with Acuson Sequoia)  n = 15 | fluoroscopy and color flow doppler  n = 15 | AF | CB | 14.5 ± 3 months | 2 |
| Dello Russo, 2015 | Prospective comparative non-RCT | 37 | sensor-based or non-sensor-based ICE catheter (AcuNav or SoundStar)  n = 20 | CARTO3, fluoroscopy and tissue connect only  n = 17 | AF  paroxysmal: n = 31 persistent: n = 6 | RF | 12 months | 3 |
| Herman, 2017 | RCT | 79 | non-sensor based ICE catheter (AcuNav with VividQ)  n = 40 | fluoroscopy  n = 39 | CTI-AFL | RF | NR | 2 |
| Khaykin, 2011 | Retrospective comparative  non-RCT | 433  (>500 procedures) | Fluoroscopy plus non-sensor based ICE catheter (AcuNav); no EAM  n = 197 | EAM (CARTO XP, CARTO3)  n = 71  EAM (NavX)  n = 165 | AF  paroxysmal: n = 273 persistent: n = 111  permanent: n = 49 | RF | fluoroscopy plus non-sensor based ICE:  2.7 ± 1.0 years  CARTO:  2.4 ± 0.7 years  NavX = 2.7 ± 1.0 years | 4 |
| Khaykin, 2009 | RCT | 60 | non-sensor based ICE catheter (AcuNav)  n = 30 | EAM (CARTO) + ablation of complex fractionated electrograms  n = 30 | AF  paroxysmal: n = 48 persistent: n = 12 | RF | 2 ± 1 year | 3^ |
| Lyan, 2018 | Retrospective comparative non-RCT | 481 | non-sensor based ICE catheter (AcuNav)  n = 245 | Fluoroscopy and EAM (CARTO3)  n = 236 | Paroxysmal AF | RF | 15.2 ± 4.1 months | 4 |
| Mah, 2014 | RCT | 84 | sensor based, CARTO-integrated catheter (SoundStar)  n = 42 | EAM (CARTO3)  n = 42 | SVT  AVNRT: n = 35  WPW: n = 31  URAP: n = 16  FAT or Mahaim: n=2 | RF | 2.1 months (median) | 2 |
| Marrouche, 2007 | RCT | 53 | non-irrigated RF catheter; non-sensor based ICE catheter (AcuNav), microbubble-guided  n = 27 | open-irrigation tip RF catheter, EAM (CARTO), ICE visualization  n = 26 | AF  paroxysmal: n = 33  persistent/permanent: n = 20 | RF | 14 ± 2 months | 3^ |
| Marrouche, 2003 | Retrospective comparative non-RCT | 315 | circular mapping + non-sensor based catheter (ICE without bubbles)  n = 107  circular mapping + non-sensor based catheter + RF titration (ICE with bubbles)  n = 152 | circular mapping  n = 56 | AF  paroxysmal: n = 160  persistent: n = 41 permanent: n = 144 | RF | 417 ± 145 days | 4 |
| Miyake, 2011 | RCT | 74 | non-sensor based ICE catheter (AcuNav)+ EAM = 37 | fluoroscopy  n = 37 | SVT | RF | NR | 2 |
| Pratola, 2011 | RCT | 60 | sensor-based, CARTO-integrated ICE catheter (SoundStar) + MRI  n = 20  sensor-based, CARTO-integrated ICE catheter (SoundStar)  n = 20 | MRI and EAM (CARTO, CARTOMerge)  n = 20 | AF  paroxysmal: n = 45  persistent: n = 15 | RF | 9.1 ± 2.2 months | 2 |
| Proietti, 2017 | Retrospective comparative non-RCT | 16  (24 procedures) | sensor-based, CARTO-integrated ICE catheter (SoundStar) + EAM  n = 15/24 procedures | EAM (CARTO3)  n = 9/24 | VT | RF | 10.5 ± 7 months | 4 |
| Razminia, 2012 | Retrospective comparative non-RCT | 120 | IE + non-sensor-based ICE catheter + EAM (no fluoroscopy)  N = 60 | fluoroscopy + EAM  n = 60 | AF: n = 44  AFL: n = 20  AT: n = 18  AVNRT: n = 20  AVNRT: n = 12  VT: n = 6 | RF | NR | 4 |
| Rubesch-Kutemeyer, 2017 | Retrospective comparative non-RCT | 100 | non-sensor based ICE catheter (AcuNav)  n = 50 | fluoroscopy  n = 50 | Paroxysmal AF | CB | non-sensor based catheter: 11.8 ± 3.1 months  fluoroscopy: 12.8 ± 3.2 months | 4 |
| Schmidt 2010 | RCT | 43 | non-sensor based ICE catheter (AcuNav)  n = 22 | fluoroscopy  n = 21 | Paroxysmal AF | CB | 6 months | 2 |

Key: *Mean follow-up presented, unless otherwise specified. ^ Level of evidence adjusted downward for possible confounding of comparison of interest

Abbreviations: AF = atrial fibrillation, AFL = atrial flutter, AVNRT = atrioventricular nodal reentry tachycardia, CB = cryoballoon, CTI = cavo-tricuspid isthmus, CT = computed tomography, EAM = electroanatomical mapping, ICE = intracardiac echocardiography, MRI = magnetic resonance imaging, NR = not reported, PV = pulmonary vein, PVC = premature ventricular contraction, RCT = randomized controlled trial, RF = radiofrequency, TEE = transesophageal echocardiography, URAP = unidirectional retrograde accessory pathway, WPW = Wolff Parkinson White Syndrome, VT = ventricular tachycardia.

Supplemental Table 3: Sensitivity analyses of fluoroscopy time (primary outcome) in meta-analysis of the use of ICE vs. comparator (no ICE) in catheter ablation of cardiac arrhythmias.

|  | **Estimate*** | **95% CI** | **No. Studies** | ***p* -value** |
| --- | --- | --- | --- | --- |
| HKSJ method using full dataset | -1.06 | (-1.78; -0.34) | 14 | < 0.01 |
| Remove 1 outlying and influential study   (Lyan, 2018) | -0.86 | (-1.36; -0.36) | 13 | < 0.01 |
| Include 3 studies with imputed mean and/or SD   values^†^ (Razminia, 2012; Mah, 2014; Miyake, 2011) | -1.05 | (-1.68; -0.42) | 17 | < 0.01 |
| Use alternate group for 3-arm studies   (Khaykin, 2011; Marrouche, 2003; Pratola, 2011) | -0.93 | (-1.80; -0.07) | 14 | 0.04 |
| Limit to studies with 100% AF patients | -1.25 | (-2.14; -0.36) | 11 | < 0.01 |
| Mean difference analysis | -8.12 minutes | (-13.45; -2.79) | 11 | < 0.01 |
| Limit to sensor-based ICE catheter  (SOUNDSTAR® Catheter) studies | -1.27 | (-2.31; -0.24) | 3 | 0.02 |
| Mean difference analysis | -12.74 minutes | (-15.60; 9.88) | 3 | < 0.01 |

*Unless otherwise stated, difference estimates are based on the DerSimonian-Laird (DL) method.

^†^The standard deviation for the ICE group in Razminia (2012) was imputed with an outcome-specific linear model regressing log (standard deviations) on log (means). The means and standard deviations for Mah (2014) and Miyake (2011) were imputed using formulas found in Hozo (2005).

Abbreviations: AF = atrial fibrillation, CI = confidence interval,
HKSJ = Hartung-Knapp-Sidik-Jonkman, ICE = intracardiac echocardiography, SD = standard deviation.

Supplemental Table 4: Sensitivity analyses of secondary efficiency outcomes in meta-analysis of the use of ICE vs. comparator (no ICE) in catheter ablation of cardiac arrhythmias.

|  | **Estimate*** | **95% CI** | **No. Studies** | ***p* –value** |
| --- | --- | --- | --- | --- |
| **Fluoroscopy dose** |  |  |  |  |
| HKSJ method using full dataset | -1.27 | (-2.15; -0.38) | 10 | 0.01 |
| Remove 1 outlying and influential study   (Marrouche, 2007) | -1.55 | (-2.01; -1.10) | 9 | < 0.01 |
| Include 2 studies with imputed mean and/or SD   values^†^ (Mah, 2014; Miyake, 2011) | -1.08 | (-1.68; -0.49) | 12 | < 0.01 |
| Limit to studies with 100% AF patients | -1.32 | (-2.04; -0.59) | 9 | < 0.01 |
| **Procedure time** |  |  |  |  |
| HKSJ method using full dataset | -0.35 | (-0.77; -0.08) | 10 | 0.10 |
| Remove 1 outlying and influential study   (Dello Russo, 2015) | -0.24 | (-0.51; 0.03) | 13 | 0.09 |
| Include 2 studies with imputed mean and/or SD   values (Bhatia, 2010; Miyake, 2011) | -0.25 | (-0.54; 0.04) | 16 | 0.09 |
| Use alternative group for 3-arm studies   (Khaykin, 2011; Marrouche, 2003;   Pratola, 2011) | -0.28 | (-0.59; 0.02) | 14 | 0.07 |
| Limit to studies with 100% AF patients | -0.43 | (-0.74; -0.13) | 12 | < 0.01 |
| Mean difference analysis | -17.96  minutes | (-30.22; -5.71) | 12 | < 0.01 |

*Unless otherwise stated, difference estimates are based on the DerSimonian-Laird (DL) method.

^†^The means and standard deviations for Mah (2014) and Miyake (2011) were imputed using formulas found in Hozo (2005).

Abbreviations: AF = atrial fibrillation, CI = confidence interval, HKSJ = Hartung-Knapp-Sidik-Jonkman, SD = standard deviation.

Supplemental Table 5: Sensitivity analyses of secondary clinical outcomes (acute success, freedom from arrhythmia, peri-procedural complications) in meta-analysis of the use of ICE vs. comparator (no ICE) in catheter ablation of cardiac arrhythmias.

|  | **RR Estimate** | **RR 95% CI** | **No. Studies** | ***p* - value** |
| --- | --- | --- | --- | --- |
| **Acute success^†^** | | | | |
| Limit to studies with 100% AF patients | 1.00 | (0.99; 1.01) | 7 | 0.86 |
| **Freedom from arrhythmia** | | | | |
| HKSJ method using full dataset | 1.04 | (0.97; 1.11) | 11 | 0.23 |
| Use alternate group for 3-arm studies*   (Pratola, 2011) | 1.03 | (0.97; 1.10) | 11 | 0.33 |
| Limit to studies with 100% AF patients | 1.04 | (0.97; 1.11) | 11 | 0.24 |
| **Peri-procedural complications (excluding venous access)^†^** | | | | |
| Use alternate group for 3-arm studies  (Marrouche, 2003; Pratola, 2011) | 0.50 | (0.30; 0.83) | 13 | < 0.01 |
| Limit to studies with 100% AF patients | 0.71 | (0.40; 1.26) | 8 | 0.24 |
| **Venous access complications^†^** | | | | |
| Limit to studies with 100% AF patients | 3.26 | (0.52; 20.38) | 2 | 0.21 |

*Difference estimate calculated using the DerSimonian-Laird (DL) method.

†Difference estimates were calculated using the Mantel-Haenszel (MH) method.

Abbreviations: AF = atrial fibrillation, CI = confidence interval, HKSJ = Hartung-Knapp-Sidik-Jonkman, RR = risk ratio.

**A**

**
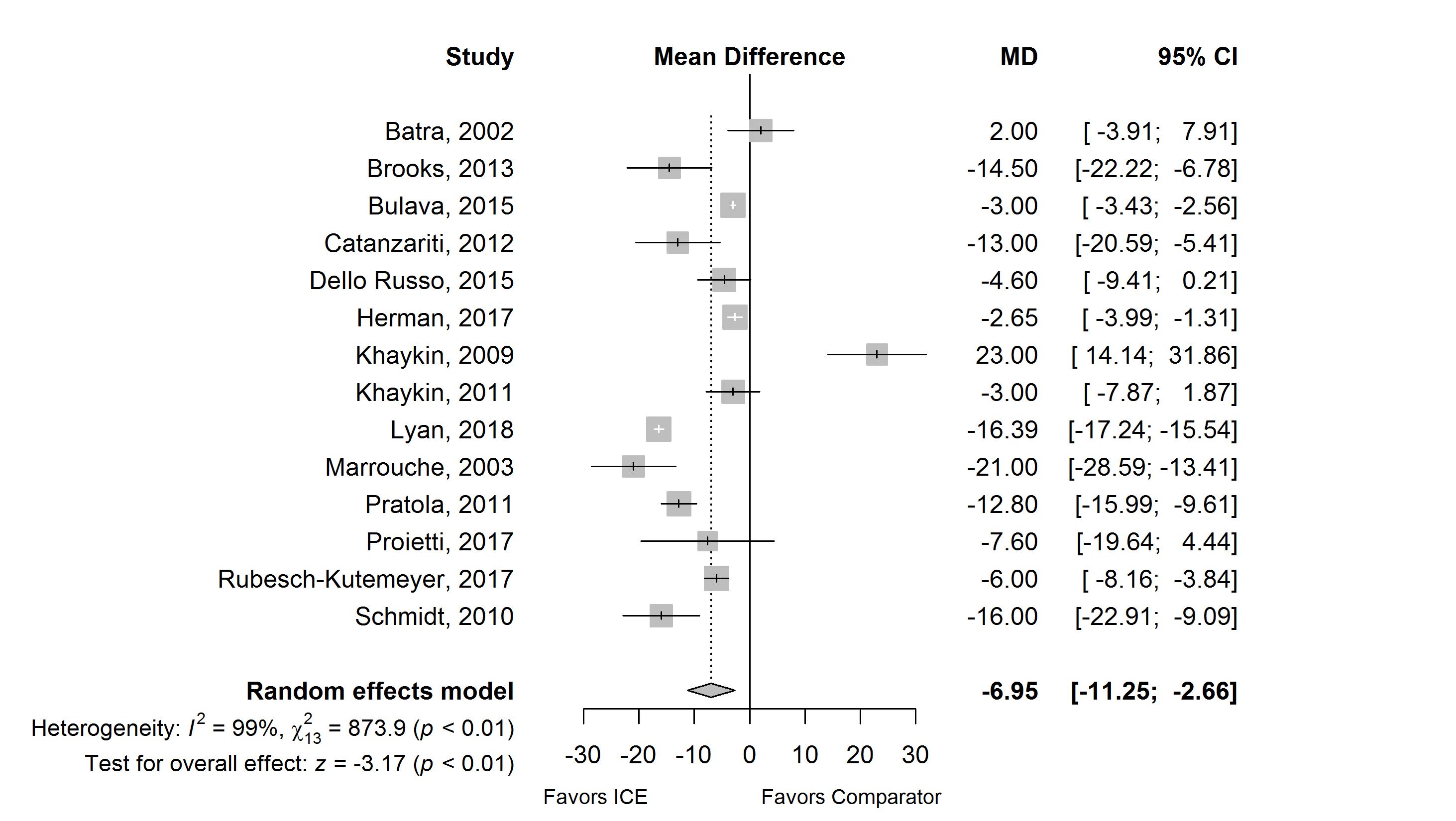
**

**B**

**
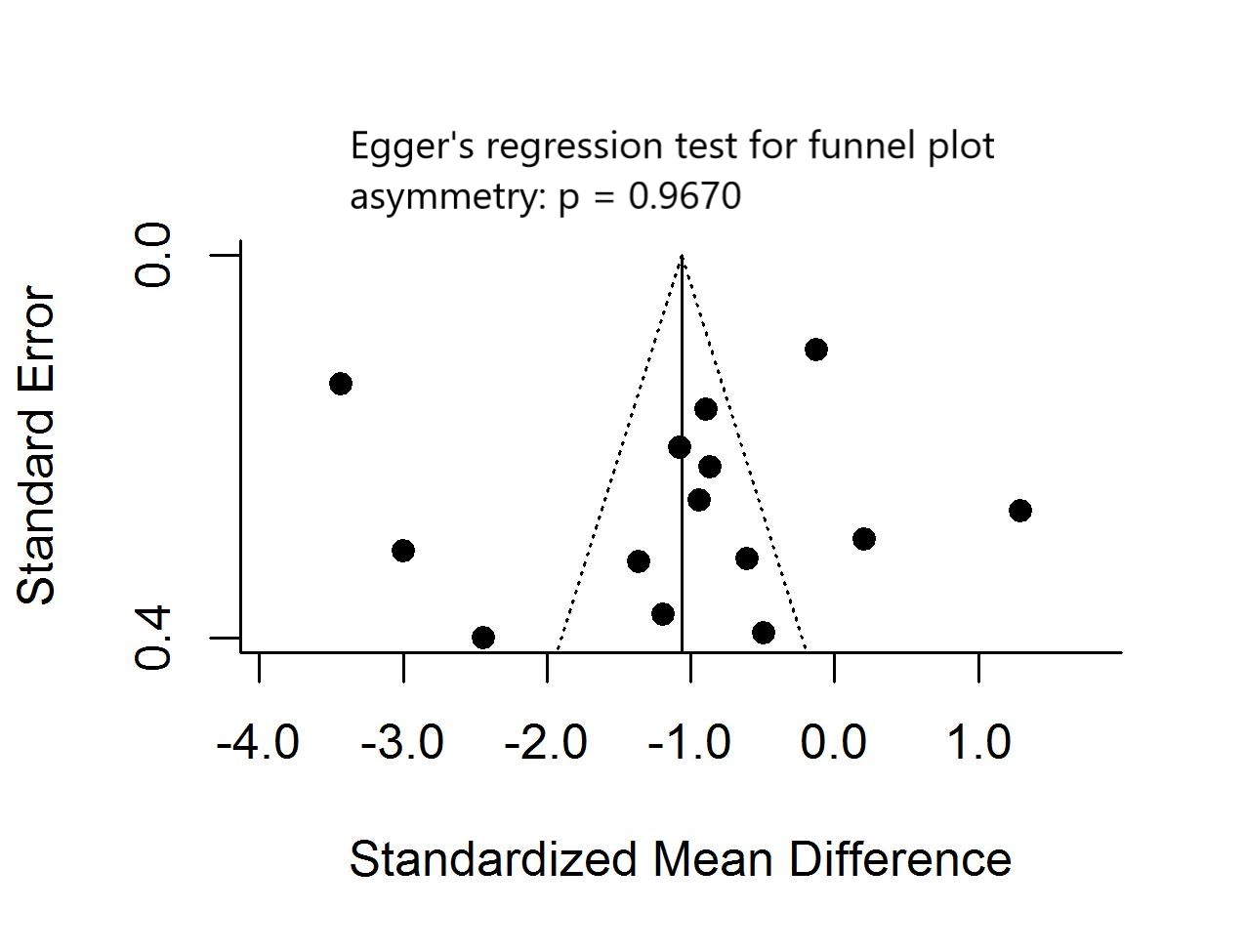
**

**Supplemental Figure 1: A) Forest plot of mean difference (minutes) analysis and B) funnel plot and regression test of publication bias for fluoroscopy time (primary outcome) in meta-analysis of the use of ICE vs. comparator (no ICE) in catheter ablation of cardiac arrhythmias.**

Abbreviations: CI = confidence interval, ICE = intracardiac echocardiography, MD = mean difference


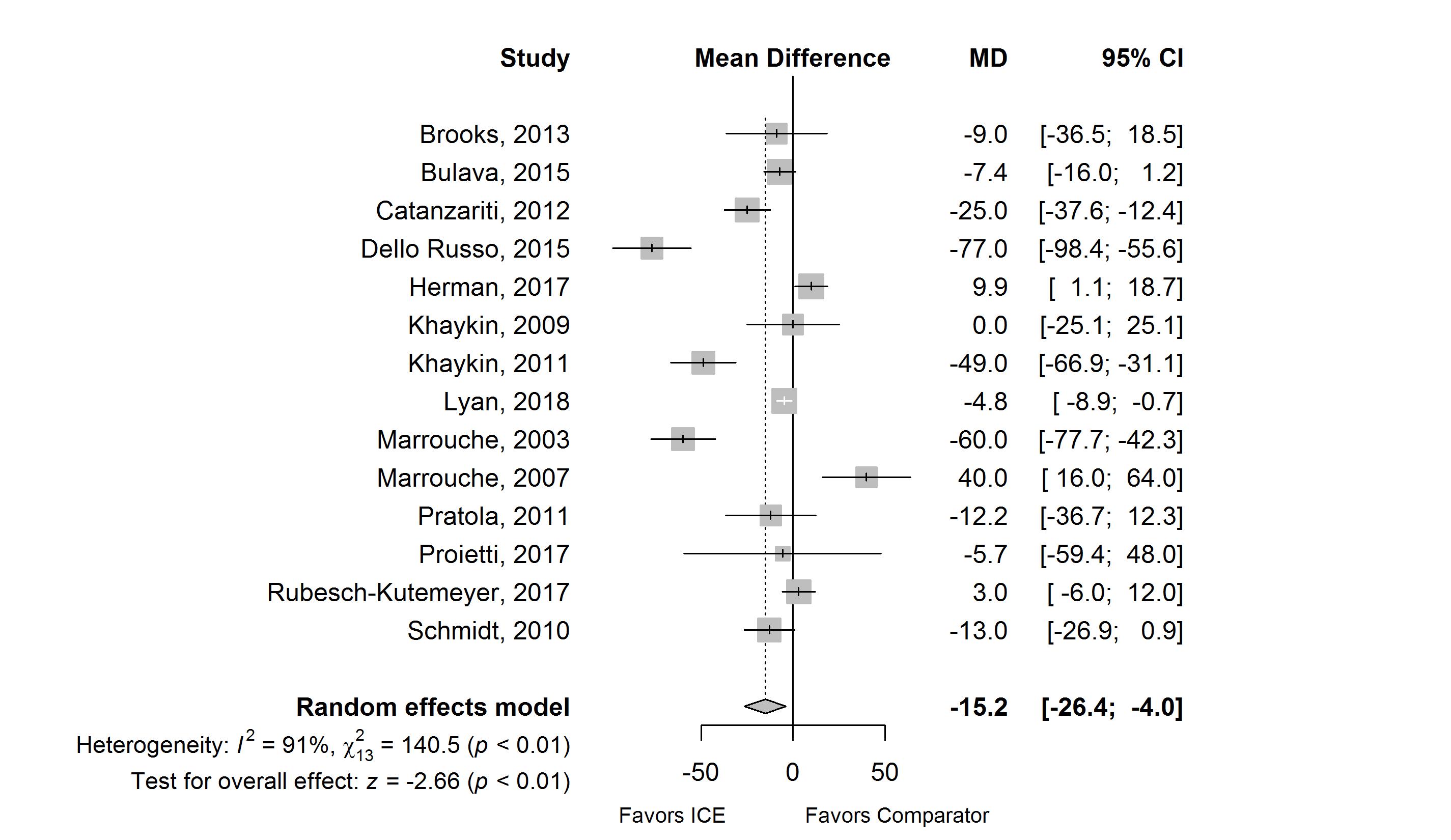


Supplemental Figure 2: Forest plot of mean difference (minutes) analysis of procedure time during catheter ablation with the use of ICE compared to ablation without ICE in arrhythmia patients.

Abbreviations: CI = confidence interval, ICE = intracardiac echocardiography, MD = mean difference

**A**


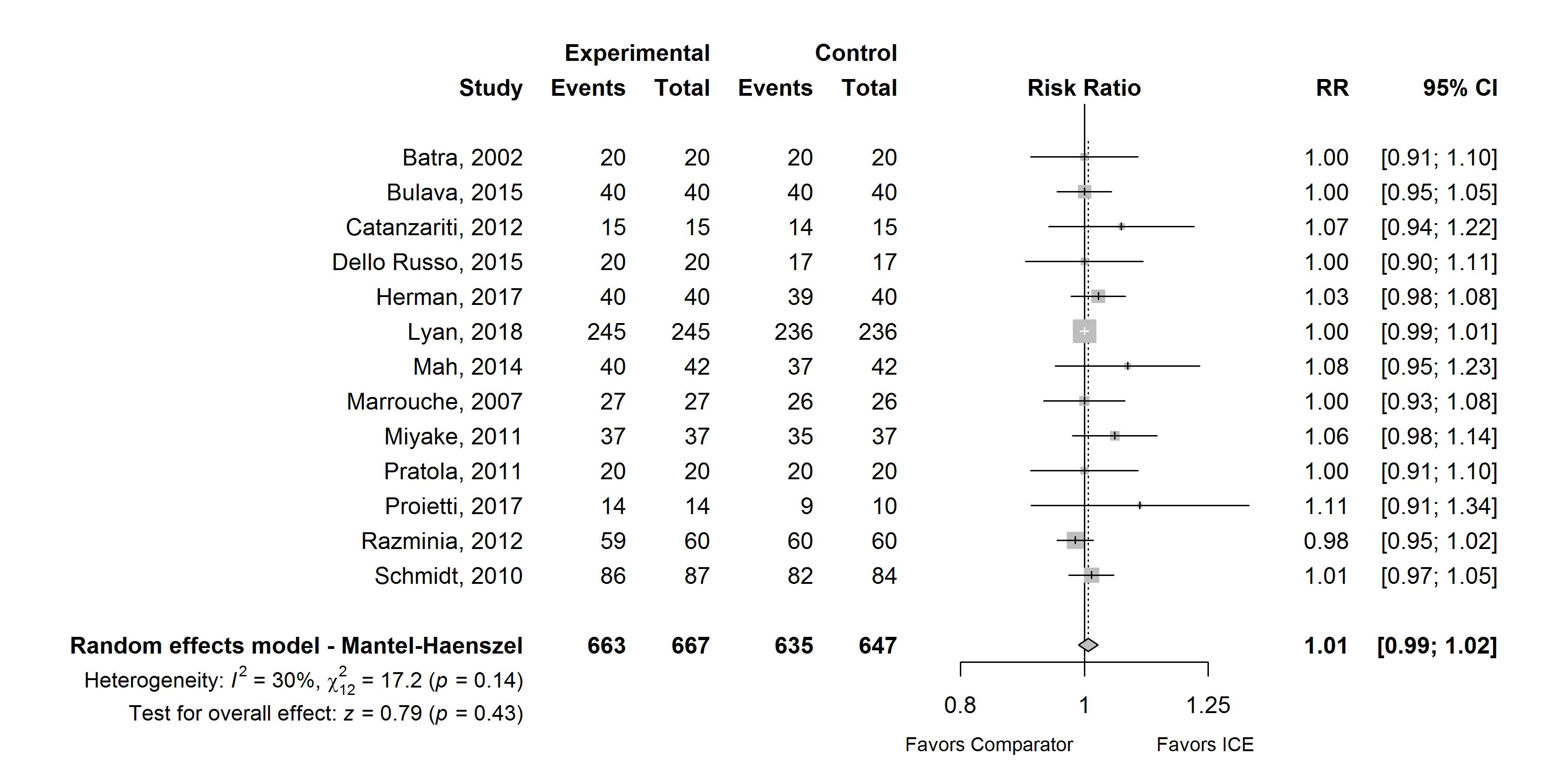


**B**


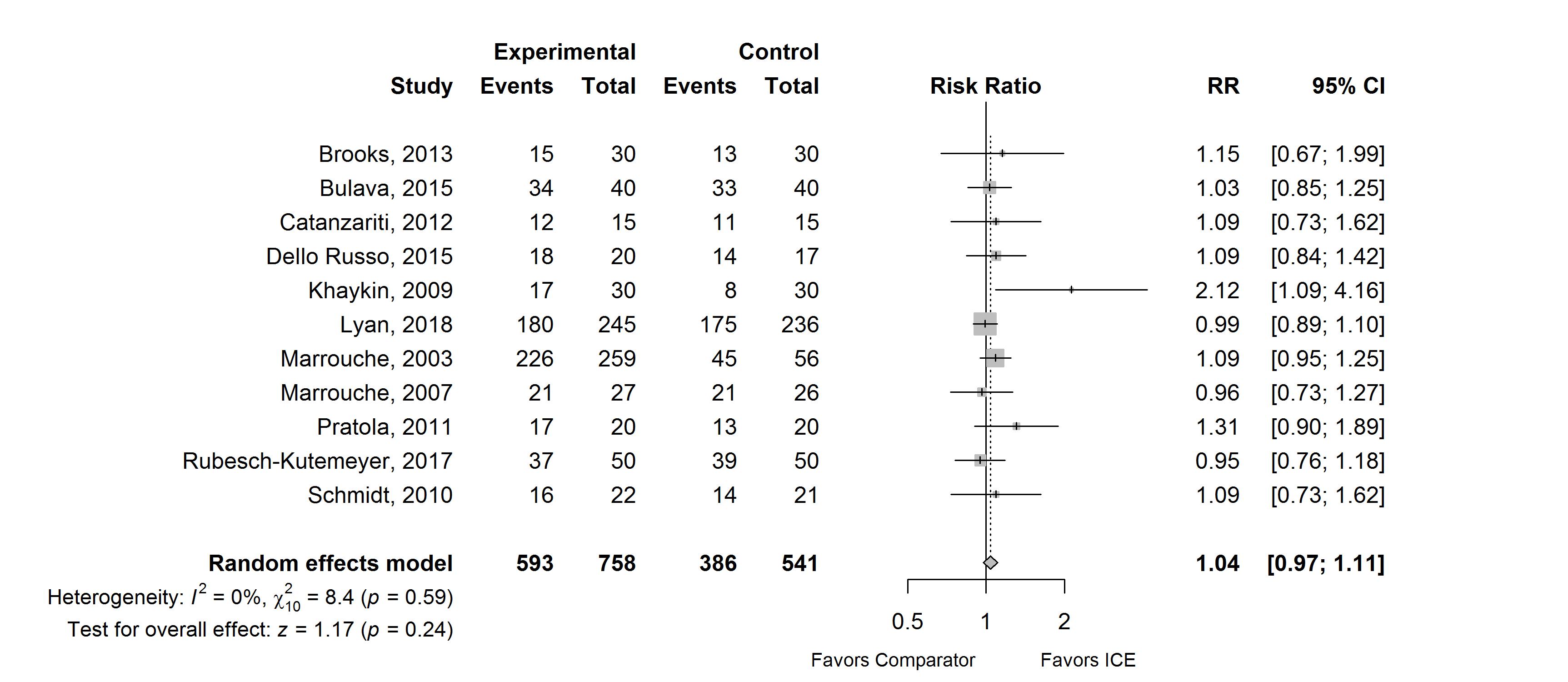


**C**


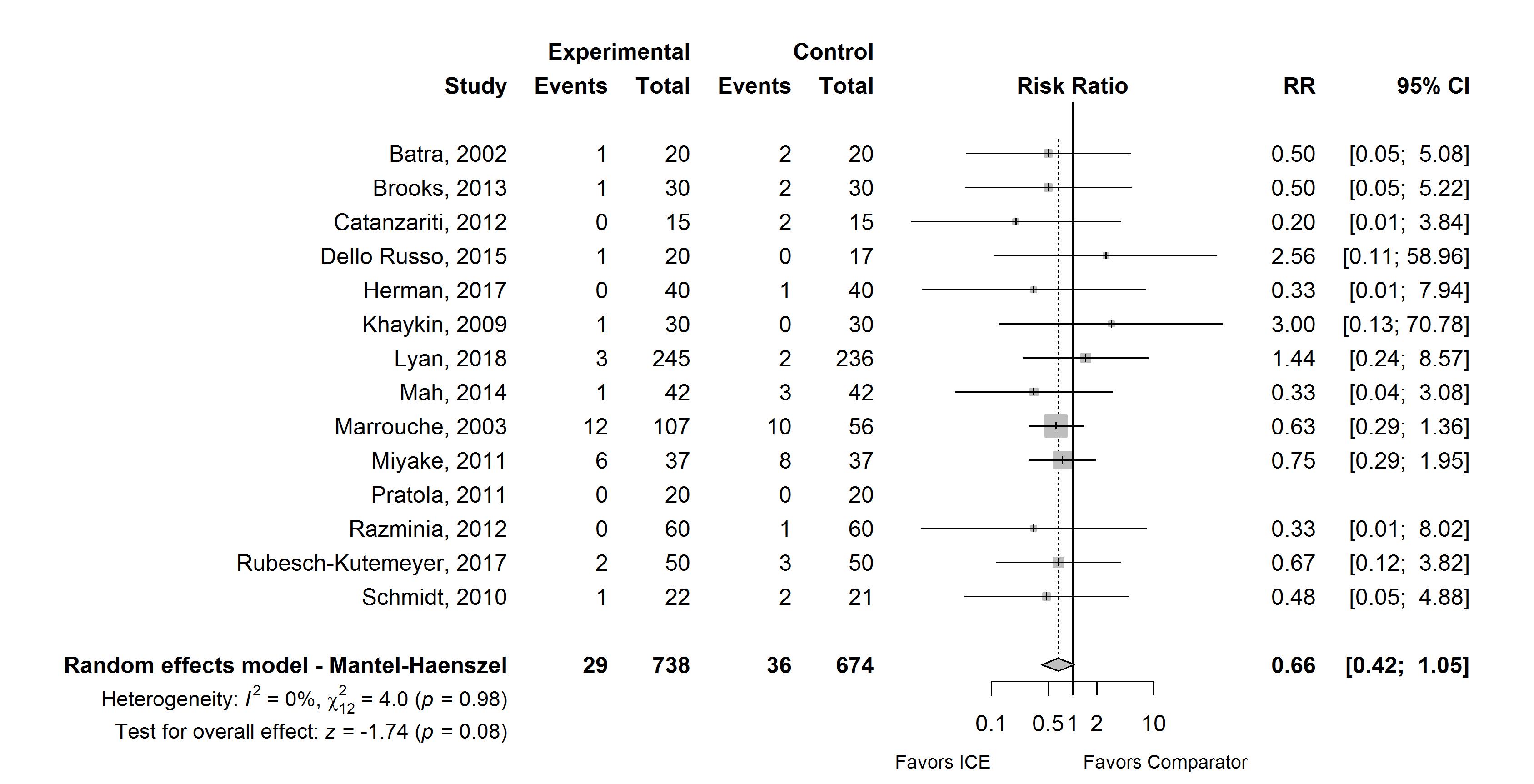


D


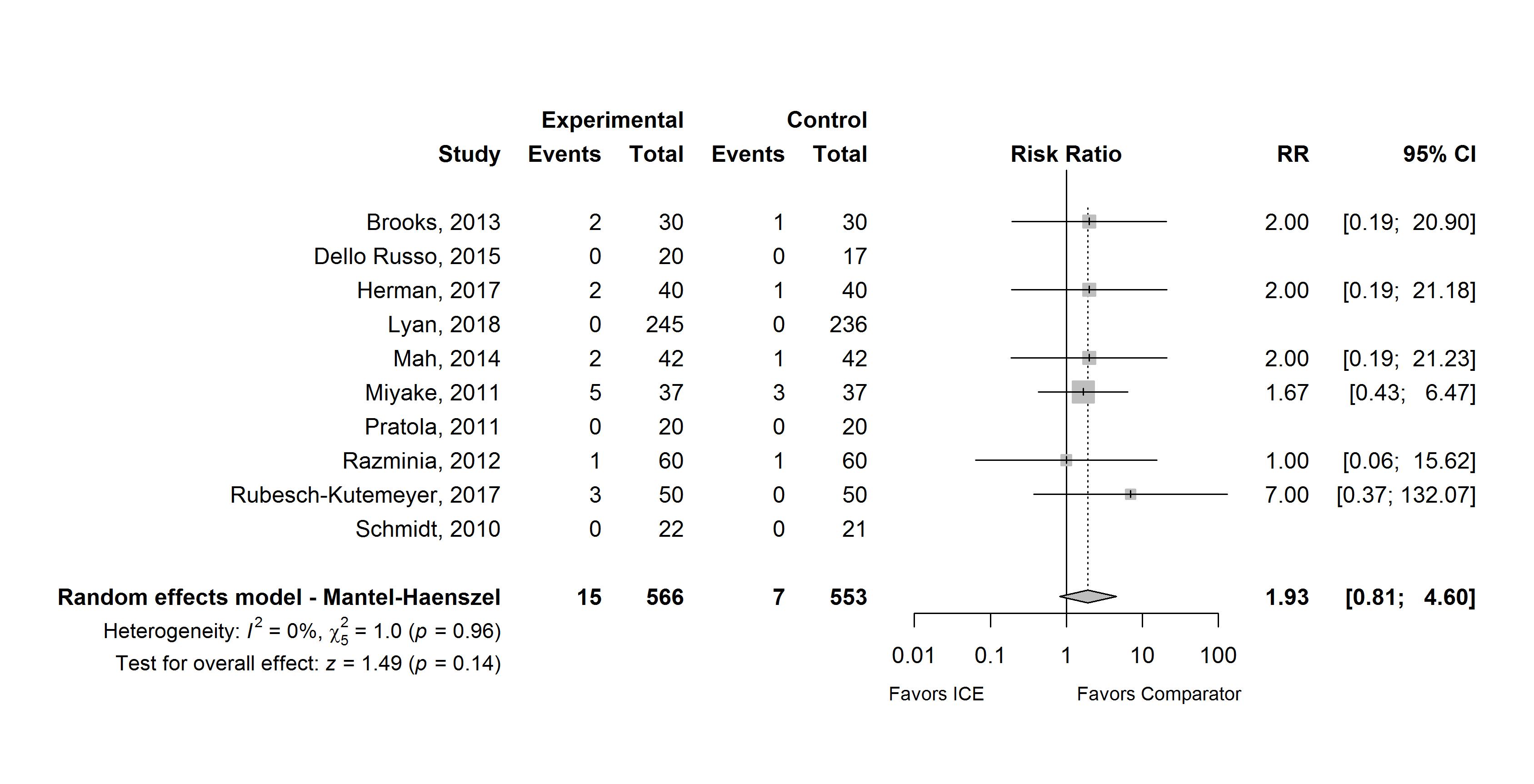


Supplemental Figure 3: Risk ratios for A) acute success, B) freedom from arrhythmia, C) peri-procedural complications (excluding venous access), and D) venous access complications outcomes in meta-analysis comparing the use of ICE vs. no ICE in catheter ablation of cardiac arrhythmias.

Abbreviations: CI = confidence interval, ICE = intracardiac echocardiography, RR = relative risk
